# Findings of a Pilot Study Investigating the Effects of Mediterranean Diet and Aerobic Exercise on Cognition in Cognitively Healthy Older People Living Independently within Aged-Care Facilities: The Lifestyle Intervention in Independent Living Aged Care (LIILAC) Study

**DOI:** 10.1093/cdn/nzaa077

**Published:** 2020-04-18

**Authors:** Roy J Hardman, Denny Meyer, Greg Kennedy, Helen Macpherson, Andrew B Scholey, Andrew Pipingas

**Affiliations:** 1 Centre for Human Psychopharmacology, Swinburne University of Technology, Melbourne, Australia; 2 Institute for Physical Activity and Nutrition, Deakin University, Geelong, Australia

**Keywords:** aging population, impact of diet, elderly, cognition, Mediterranean diet, exercise, blood markers

## Abstract

**Background:**

Cognitive decline and Alzheimer disease are more prevalent in our aging population. Modifiable risk factors, such as diet and sedentary lifestyle, have been proposed as key to potentially ameliorating cognitive decline. Both exercise and Mediterranean diet (MedDiet) have been linked to reduced levels of cardiovascular disease and other comorbidities. Higher levels of exercise and MedDiet adherence may prove to be cognitively protective, both individually and synergistically.

**Objectives:**

The aim was to investigate the effect of a 6-mo program of MedDiet, exercise, and a combination of both, on cognition, mood, and general health in older persons living independently in aged-care communities.

**Methods:**

The Lifestyle Intervention in Independent Living Aged Care (LIILAC) Study (ACTRN12614001133628) involved 102 participants, aged 60–90 y, who were randomly assigned to 1 of 4 intervention groups. Change in overall memory performance was assessed as the primary outcome. Additionally, changes in cognitive task performance, as well as mood, wellness, cardiovascular function, and blood biomarkers, were investigated.

**Results:**

While there was no significant change in overall memory performance, there was a significant improvement in spatial working memory performance in the combined exercise and diet group, relative to controls. This combined intervention group also showed an overall improvement in their emotional state, as assessed by the Depression Anxiety Stress Scale, as did the exercise-only group.

**Conclusions:**

This research indicates that diet and exercise programs have the potential to improve aspects of cognition and mood in an aging population. However, given the lower than optimal sample size and lack of resources to reinforce the interventions during the trial, further larger randomized controlled trials are required to substantiate whether the introduction of diet and exercise programs into independent-living facilities is a viable method to preserve cognitive health in older people. This trial was registered at www.ANZCTR.org.au ACTRN 12614001133628 (LIILAC Study)

## Teaser

This pilot study investigated the effect of a six-month lifestyle intervention program of diet, exercise, and a combination of both, on cognition, mood, and general health in older persons living independently. This unique study was conducted in 15 independent living facilities with testing and administration of interventions to 102 participants aged 60–90 within their own living environments.

This research indicated that diet and exercise programs have the potential to improve aspects of cognition and mood in an aging population. This study has informed a larger study, currently underway, investigating effects of MedDiet and walking program on cognitive decline and general health in a similar demographic.

## Introduction

In Australia, as in the rest of the Western world, the average life expectancy has increased (1). Progressive aging of the population has led to an increase in the number of people living with age-related neurological disorders, such as Alzheimer disease (AD), characterized by cognitive deficits (2, 3). It has been estimated that, in 2015, 47 million people around the world were living with various forms of dementia, and this is expected to increase to 75 million by 2030 and potentially to 135 million by 2050 (4). Currently, there are no effective medical treatments to alleviate the accelerated cognitive decline leading to AD. The increased incidence of AD has placed substantial financial demands on health and other related services. Of note, the aged-care sector in Australia is experiencing an increasing demand for permanent residential aged care, with >50% of permanent residential aged-care residents living with dementia (2).

Cognitive aging research indicates that there is a general increase in intellectual and cognitive abilities in infancy and adolescence, and a decline thereafter throughout the adult life span (5). The extent to which cognitive faculties decline in adulthood is variable and partly attributable to modifiable factors such as diet and exercise (5, 6). Preventative strategies utilizing simple behavioral and lifestyle changes may provide an effective means to improve cognitive function in the short term and to potentially influence cognitive trajectories over the longer term (7).

Modifiable risk factors for cognitive decline parallel those for cardiovascular and metabolic disorders (8). Risk factors such as high blood pressure, elevated cholesterol, and obesity are prevalent in Western society and are the result of poor dietary habits and a sedentary lifestyle (9). A Western diet high in saturated fats and sugar, with high amounts of processed food, can result in chronic inflammation that can adversely influence cardiovascular and metabolic function (10). Furthermore, a diet high in processed foods can lead to higher oxidative stress, leading to chronic disorders such as diabetes, which, in turn, can also affect cognitive function and decline (11).

The Mediterranean-style diet (MedDiet) is rich in fresh fruit and vegetables and whole grains, and uses olive oil as the primary source of fat. This is combined with low consumption of red meat and moderate alcohol consumption with meals (12, 13). This dietary profile has been shown to be associated with better health outcomes, including reduced incidence of cancer and cardiovascular disease (12, 14). The MedDiet is considered to possess anti-inflammatory properties that can reduce the risk of cardiovascular disease and other chronic diseases such as diabetes and metabolic syndrome (15, 16). Similar risk factors have been described for cognitive aging and chronic endpoints of aging, such as AD. More recent converging literature has shown that adherence to a MedDiet may improve cognition or reduce the risk of cognitive decline leading to AD (15–17).

Converging literature has also considered relations with exercise and cognitive decline, with findings understandably positive given the overlap between cognitive health and cardiovascular and metabolic health. A recent meta-analysis demonstrated that physical exercise is effective in improving cognitive function in older adults, independently of their cognitive status prior to initiating an exercise program (18). Improvements in cognition have also been attributed to a number of other modifiable risk factors, as identified in a recent review (19).

Two major lifestyle factors, diet and exercise, may consequently have both separate and synergistic actions on cognition and cognitive decline. Within the literature there have been a number of lifestyle studies that have evaluated how diet and exercise have been successfully utilized for cardiovascular risk reduction, yet very few controlled trials have evaluated the effects of both diet and exercise as a lifestyle intervention aimed at improving cognitive functioning and/or reducing the rate of cognitive decline, as outlined in a number of reviews (20–22).

This study aimed to investigate the effect of a change to a MedDiet and/or an increase in walking-based exercise on cognition over 6 mo. It was hypothesized that speed of memory in both the exercise and dietary intervention groups would improve relative to controls, and that the combined diet and exercise intervention would show the greatest improvement.

The study also aimed to investigate other potential markers or mechanisms of cognitive aging that may be related to diet and exercise, such as mood, wellness, cardiovascular function, and blood biomarkers.

## Methods

Detailed methodology has been previously published in the Lifestyle Intervention in Independent Living Aged Care (LIILAC) protocol (23).

### Subjects

Based on the protocol, the study aimed to recruit 148 participants aged 60–90 y, living independently in retirement and independent-living facilities, in and around Melbourne, Australia. In our experimental protocol (23) we originally indicated that a total sample of 128 participants would be required to detect a moderate effect size (η^2^ = 0.30), a power of 80%, and a significance level of 5% with 37 individuals randomly assigned into each arm of the study. However, this was not achieved. Initially, 102 participants were recruited at baseline and 81 remained at 6 mo. Written informed consent was obtained from all the participants in the study prior to their participation.

### Ethical clearance and registrations

Ethical approval was granted by Swinburne University under SUHREC 2013/057 (Swinburne University of Technology). The Australia and New Zealand Trial Registry number is ACTRN12614001133628. The universal trial number is U1111–1161-5364 (http://www.anzctrorg.au).

### Inclusion criteria

Participants were required to be fluent in written and spoken English. Those who were taking vitamins, minerals, and/or herbal supplements as part of their dietary regime were asked to continue their use for the duration of the trial, whereas those who were not taking any such supplements were requested to refrain from consuming these during the trial. Participants were also required to obtain approval from their general practitioner confirming that they were medically fit to participate in the trial and to continue taking any medications prescribed by their doctors during the trial.

### Exclusion criteria

Participants were excluded from the trial if they had any significant visual impairment that would preclude them from reading a computer screen, a significant neurological or psychiatric disorder, or were unable to walk independently and safely. Individuals were not eligible if they were using illicit drugs or cognitive-enhancing medications. Those participants with a suspected cognitive impairment [defined as a score <24 on the Mini Mental State Examination (MMSE)] or a significant number of symptoms of depression (defined as a score >9 on the Geriatric Depression Scale) were also excluded.

### Group allocation

This was a randomized, controlled, 2 × 2 factorial parallel-group study. The randomization sequence was created by permuting into blocks of 4. For reasons of practicality, and to avoid cross-contamination between the requirements of each group, cohabiting couples were allocated to the same group. Due to the nature of the interventions this study was not blinded; both investigators and participants knew which intervention group they were allocated to, as participants were required to be actively working toward maintaining the lifestyle change or, in the case of the control group, maintaining their current lifestyle, for a period of 6 mo. Participants were randomly allocated to 1 of the following 4 groups: *1*) walking intervention (exercise), *2*) adherence to a MedDiet intervention (diet), *3*) combined walking and diet intervention (exercise and diet), or *4*) control (maintenance of their current lifestyle).

### Intervention

The exercise group was requested to walk 30–40 min/d following a lead-in of 15 min/d, which was gradually increased over the first month. The distance covered was monitored via a pedometer. Participants were also requested to keep a log of their steps and the distance covered, which served to ensure compliance and was used as a motivational tool.

The diet groups received a collection of recipes to assist with preparing meals in keeping with a Mediterranean dietary style, which incorporates extra-virgin olive oil (EVOO) as the primary source of fat. The recipes for breakfast, morning tea, lunch, afternoon tea, dinner, and dessert were included in a printed 6-wk MedDiet meal plan that was prepared for the trial with the assistance of dietary consultants from Health Care 2 U.

As this was an unfunded study, the participants were initially counseled regarding the MedDiet and how to change to a MedDiet style; however, no dietitian or food training was possible. The diet intervention was standardized across all participating sites. While it was recommended that participants follow the 6-wk plan, they could also choose specific recipes according to their preference. Participants were counseled on the use of this dietary plan by the researchers at baseline and then at the follow-up interview at 3 mo to ensure they were on track. To assist with daily meal preparation, participants received EVOO provided free of charge by Cobram Estate. Participants were requested to only use this EVOO and not to use any other cooking or baking oils.

Participants were asked to score their adherence to a MedDiet as described in the recipe book; at the end of each day, they provided a score from 1 to 4 indicating their adherence to the dietary intervention. These scores enabled the assessment of compliance to a MedDiet.

The exercise and diet group was requested to follow the interventions of both the exercise and diet groups as described above. The control group was requested to maintain their lifestyle over the 6-mo trial period.

### Diet assessment/MedDiet score

Habitual diet was assessed using the Cancer Council of Victoria Dietary Questionnaire [food-frequency questionnaire (FFQ)] for Epidemiological Studies, version 2 (November 2014) (24), both at baseline and following the 6-mo intervention period. The output of the FFQ was utilized to produce a MedDiet score (MedDietS) in accordance with Trichopoulou et al. (25). A sex-specific median of dietary intake across all participants in the study was calculated at baseline, allowing for a comparative cutoff to be made for each sex on food consumption. Foods classed as belonging to the MedDiet, such as vegetables, legumes, fruits, nuts, cereals, and fish, were assigned a value of 0 if a person's consumption was below the sex-derived median, and a score of 1 if it was equal to or above, the median. For non-MedDiet food components, such as meat, poultry, and dairy, consumption above the median was scored as 0 and intake below the median was scored as 1. For alcohol, a score of 1 was given, provided consumption was within 10 and 50 g/d for men and 5 and 25 g/d for women. When considering fat intake, the ratio of monounsaturated to polyunsaturated lipids was evaluated, with a ratio above the median being allocated a 1 and below the median as 0. The total MedDietS ranged from 0 (minimal adherence to the traditional MedDiet) to 9 (maximal adherence).

### Assessment of walking ability

The 6-Minute Walk Test (6MWT) was used to determine how far an individual could walk in 6 min. This was conducted at baseline and at 6 mo. The 6MWT has been shown to be a valid and reliable test of physical walking ability that has been related to aerobic fitness (26). The 6MWT was performed along a flat, straight, hard surface inside or outside the testing facility. The walking course was 10 m in length. The objective of this test is to walk back and forth along the course as many times in 6 min.

### Cognitive assessment

The assessment of cognitive performance utilized the Swinburne University Computerized Cognitive Assessment Battery (SUCCAB). The SUCCAB is a validated computer-based cognitive battery consisting of 8 measures that focus on the cognitive domains that decline with advancing age: simple and choice reaction times (CRTs), immediate recognition memory (IRM), delayed recognition memory (DRM), congruent and incongruent Stroop color-words, spatial working memory (SWM), and contextual memory (CM) (5). Mean correct response times and percentage of response accuracy for each measure were automatically calculated by the SUCCAB system.

Additionally, overall performance on each task was assessed by dividing accuracy percentage by the mean response time for correctly performed trials. This calculation serves to reduce the number of measures, as well as accounting for any speed/accuracy trade-off in performance present in this older group (27–31).

### Primary outcome

The primary outcome, as defined in the LIILAC protocol paper (23), was the overall change in memory response times, calculated as a composite measure from the SUCCAB memory tests. This measure, the composite memory measure (CompM), was calculated as the sum of the participant's mean IRM, DRM, SWM, and CM task response times. Change was then calculated as the difference in CompM scores between baseline and final assessment session at 6 mo.

### Secondary outcomes

These included the effects on the extended SUCCAB cognitive performance measures (performance = accuracy/reaction time), mood, wellness, peripheral and central blood pressure, arterial stiffness, and blood biomarkers of inflammation, gluco-regulation, cholesterol, brain-derived neurotropic factor (BDNF), and homocysteine, as well as concentrations of vitamins D, B-6, and B-12.

### Demographic and anthropomorphic measures

Age, sex, and educational status were recorded. Age (in years) was calculated from self-reported birth date. Highest educational level completed was reported as school (junior or high), tertiary, or postgraduate. Height, weight, hip, and waist circumference were measured and recorded. BMI (kg/m^2^) was computed. The hip-to-waist ratio was computed using physical measurements of both the hips and waist (see **Table 1**).

**TABLE 1:** Baseline characteristics of the groups^1^

Group	*n*	Age, y	BMI, kg/m^2^	HWR, cm:cm	Systolic BP, mm Hg	Diastolic BP, mm Hg
Exercise	26	77.50 ± 7.44	29.1 ± 4.69	1.07 ± 0.09	138.04 ± 17.40	71.60 ± 11.08
Diet	25	77.68 ± 7.38	28.26 ± 5.37	1.07 ± 0.08	134.76 ± 16.83	69.32 ± 11.03
Exercise and diet	24	76.54 ± 7.37	26.64 ± 3.39	1.07 ± 0.10	133.00 ± 12.17	68.21 ± 8.17
Control	27	78.22 ± 5.81	29.01 ± 4.90	1.06 ± 0.07	136.89 ± 16.92	72.04 ± 10.56

^1^Values are means ± SDs unless otherwise indicated. BP, blood pressure; HWR, hip-to-waist ratio.

### Mood and wellness assessment

The Depression Anxiety Stress Scale (DASS) was used to assess changes in mood across the study. The DASS has 3 related negative emotional states of depression, anxiety, and stress and a total composite score is also calculated (32). A lower total DASS score represents fewer combined negative symptoms.

The Profile of Mood States (POMS) was used to assess transient, fluctuating feelings and enduring affect states over the past week (33). It produces scores for 6 dimensions of mood (anger, anxiety, confusion, vigor, fatigue, and depression). A total “mood disturbance” score was derived.

The perceived-wellness survey is a set of statements that are designed to provide information about a person's wellness perceptions. The survey is a multidimensional measure of sublevels that consider perceived-wellness perceptions in relation to an individual's physical, spiritual, psychological, social, emotional, and intellectual dimensions. These scores can be evaluated separately. In this research, we report a composite perceived-wellness score (PWS), with higher scores indicating greater perceived wellness (34).

### Cardiovascular assessment measures

Peripheral and central blood pressures, as well as measures of arterial stiffness, were assessed using the SphygmoCor® device (Model XCEL; AtCor Medical), with each participant in the supine position lying on their back with the left arm placed in line with the height of the heart (a pillow or support was used to achieve this). Participants were requested to remain in the supine position for 5 min prior to measurement. For the last 2 min of the resting period and during the blood pressure assessment, the participant was instructed not to talk as this may affect the reading. Standard brachial blood pressure was measured by taking 3 brachial pressure cuff assessments over the left arm. The first recording was discarded and the average of the second and third recordings was used. Second, the SphygmoCor device was used to calculate central (aortic) blood pressure and measures of arterial stiffness, augmentation pressure (AP) and augmentation index, using central arterial pressure waveform analysis (35). Central pulse pressure was derived as the ratio of central systolic to central diastolic blood pressure.

Pulse-wave velocity (PWV) is considered to be the gold-standard measure of arterial stiffness and is derived using a femoral cuff to capture the femoral waveform and tonometer pressure sensor to capture the carotid waveform. PWV is calculated as the distance between the carotid and femoral measurement sites divided by the transit time (36, 37).

### Blood biomarker assessments

During the consultation with their medical practitioner, each participant was given a pathology request to attend Dorevitch Pathology for blood collection. Blood results were accessed by researchers and the medical practitioner via a secure Web-based portal organized by Dorevitch Pathology. The blood markers analyzed were as follows: cholesterol; glucose; high-sensitivity C-reactive protein (hsCRP); vitamins B-12, B-6, and D; homocysteine; glycated hemoglobin (HbA1c), BDNF, and insulin-like growth factor I (IGF-I; somatomedin C). All blood samples were evaluated using automated pathology equipment by Dorevitch Pathology, except for BDNF, which was assessed in Swinburne University laboratories using a sandwich ELISA kit (catalog no. CYT306).

### Statistical analysis

Sex, age, and education were predicted to be associated with cognition, cardiovascular variables, mood, and biomarkers, and were therefore used as control variables in **Tables 2** –**5** in all group comparisons (38, 39).

**TABLE 2:** Group comparisons for cognition measures with the control group as the reference group^1^

Outcome	CompM	ST	CRT	IR	DR	ConS	InconS	SWM	CM
Baseline measure	0.48***	−0.773***	0.46***	0.49***	0.73***	0.60***	0.67***	0.73***	0.76***
Age	2.39	−0.01	0.04	−0.03	−0.05*	0.06	−0.01	−0.03	−0.01
Male	25.55	−0.93	1.39**	0.39	−0.19	1.61*	−0.67	0.46	−0.29
School (junior or high)	−64.69	−1.58	−0.08	−0.09	−0.35	−0.08	−0.24	0.76	−0.05
Tertiary education	−61.03	−2.34	−0.09	0.05	−0.78	−0.09	−0.92	0.85	0.29
HcY	−8.78	0.18	−0.10	0.03	−0.02	−0.12	−0.07	0.04	0.03
Exercise group	−4.34	−0.31	−0.82	−0.22	0.28	−0.51	−0.57	0.59	0.03
Diet group	−36.62	0.02	−0.26	0.22	0.31	0.24	−0.54	0.22	0.27
Exercise and diet group	−52.39	1.64	−0.04	0.65	0.05	−0.18	0.00	0.87*	0.58
Group effect size (η^2^)	0.01	0.05	0.03	0.05	0.01	0.01	0.05	0.07	0.02

^1^Values were controlled for the corresponding baseline measure, age, sex, education, and baseline homocysteine concentrations (unstandardized coefficients). *P < 0.05, ** *P* < 0.01 ***P < 0.001. CM, contextual memory; CompM, composite measure reaction time; ConS, congruent Stroop; CRT, choice reaction time; DR, delayed recognition; HcY, homocysteine at baseline; InconS, incongruent Stroop; IR, immediate recognition; ST, simple reaction time; SWM, spatial working memory.

Baseline comparisons of the groups (using the control group as the reference category) were conducted using ANOVAs. Next, using age, sex (with female as the reference category), highest educational level (with postgraduate education as the reference category), and any baseline measures that differed significantly between the groups as control variables, ANCOVAs were conducted to test for significant differences between the groups in terms of the above outcome measures at 6 mo, with the control group regarded as the reference category in all cases.

All analyses were conducted using SPSS Statistics, version 24 (SPSS, Inc.), and *P* values < 0.05 were regarded as significant. Effect sizes (η^2^) of between 0.06 and 0.14 were regarded as moderate effects, with effect sizes of η^2^ > 0.14 regarded as large effects.

## Results

### Recruitment, allocation, and profile of participants

**FIGURE 1:**
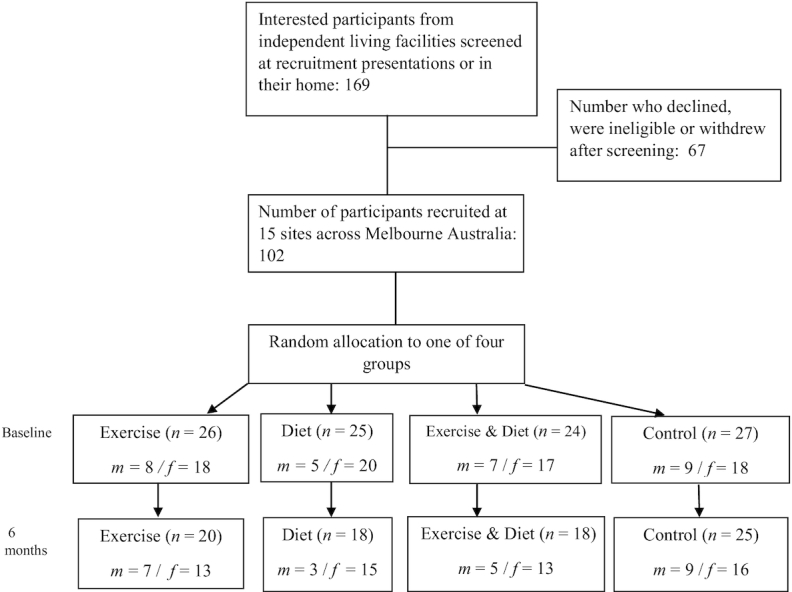
Recruitment, allocation, and attrition of participants. f, female; m, male.

**Figure 1** shows the number of participants, their sex, and the breakdown of groups within the trial from initial recruitment to assessment at 6 mo.

The intended sample size (148) was not met with 102 participants recruited at baseline. Additionally, although we predicted a 15% attrition rate, the actual rate was higher than anticipated at 20.5%, resulting in 81 participants remaining at the conclusion of the trial.

There was a 23% attrition in the exercise group, 28% within the diet group, and 25% within the exercise and diet group, and a 7% loss within the control group between the time of initial recruitment and the 6-mo assessment. However, there was no significant difference in attrition rates between the groups (chi-square = 3.26, *df* = 3, *P* = 0.353). The loss of participants was the result of individuals not being able to maintain a commitment to the trial and changes in personal circumstances. Baseline characteristics of the groups are presented in Table 1.

There were no significant differences between the groups in terms of mean age [*F*(3,98) = 0.25, *P* = 0.861] or sex (chi-square = 2.243, *df* = 3, *P* = 0.523).

A table of complete baseline results including cognitive, mood, and wellness measures; cardiovascular measures; and biomarkers can be found in the **Supplemental Appendix**. This table shows no significant difference between the groups for any measure at baseline (T1) except for homocysteine, which was particularly low for the diet group. Homocysteine is therefore included in the ANCOVAs as a covariate.

The groups were compared in terms of the outcome measures at 6 mo while controlling for the baseline values for each outcome measure, age, sex, highest educational level, and baseline homocysteine. The reference categories were female in the case of sex, postgraduate in the case of educational level, and the control group in the case of group analysis. Parameter estimates are shown in all of the ANCOVA tables with effect sizes (η^2^) displayed only in the case of the group effect.

### Cognitive assessment

Table 2 shows the ANCOVA results for the CompM reaction time as well as the secondary measures of cognitive performance [accuracy (%)/reaction time (s)]. There were no significant group effects for the primary CompM.

For the extended SUCCAB cognitive measures, the exercise and diet intervention group demonstrated significantly better SWM performance (*P* < 0.05, η^2^ = 0.06) than the control group after 6 mo.

In addition to cognitive changes for the groups, there were a number of significant associations with control variables. There was a significant decline in DRM with advancing age (*P* < 0.05, η^2 ^= 0.06). Males performed better than females in relation to CRT (*P* < 0.01, η^2 ^= 0.10) and congruent Stroop (*P* < 0.05, η^2^ = 0.10).

### Mood and wellness assessment

Table 3 shows the ANCOVA results for depression, anxiety, and stress from the DASS, the total DASS, total POMS, and PWS. The exercise group and the exercise and diet group demonstrated a significantly lower total DASS score than the control group (*P* < 0.05 and η^2^ = 0.10 and *P* < 0.05 and η^2^ = 0.09, respectively) at 6 mo. However, the exercise and diet group showed significantly reduced PWS as compared with the control group (*P* < 0.05, η^2^ = 0.07).

**TABLE 3:** Group comparison for mood and wellness measures with the control group as the reference group^1^

Outcomes	Depression	Anxiety	Stress	Total DASS	Total POMS	PWS
Baseline measure	0.19***	0.15***	0.48***	0.95***	0.71***	0.08
Age	0.03	0.00	0.00	0.09	0.32	0.12
Male	−0.42	−0.36	0.23	0.59	3.01	−1.24
School (junior or high)	−0.20	0.02	−0.33	−0.66	6.55	−2.05
Tertiary education	0.61	−0.25	0.19	−0.78	1.91	−2.30
HcY	0.07	−0.03	−0.06	−0.03	−0.57	−0.13
Exercise group	−0.17	0.24	0.75	−2.76*	−7.19	−1.65
Diet group	0.37	0.17	−0.31	−1.13	−0.74	−1.24
Exercise and diet group	−0.31	−0.38	−0.57	−2.63*	−8.80	−2.64*
Group effect size (η^2^)	0.03	0.04	0.04	0.13	0.08	0.07

^1^Values were controlled for the corresponding baseline measure, age, sex, education, and baseline homocysteine concentrations (unstandardized coefficients). *P < 0.05, ***P < 0.001. DASS, Depression Anxiety Stress Scale; HcY, homocysteine at baseline; POMS, Profile of Mood States; PWS, perceived-wellness score.

### Cardiovascular assessment measures

Table 4 demonstrates that there were no significant differences between the groups and the control group with regard to cardiovascular assessments. Higher homocysteine concentrations were associated with significantly higher PWV (*P* < 0.05, η^2^ = 0.07) and there was significantly lower AP for males than females (*P* < 0.05, η^2^ = 0.10).

**TABLE 4:** Group comparisons for cardiovascular assessment with control group as the reference group^1^

Outcomes	Systolic	Diastolic	PWV	PP	AP	AIx
Baseline measure	0.57***	0.44***	0.70***	0.59***	0.32***	0.86
Age	0.30	0.00	0.02	0.25	0.20	0.99
Male	−2.11	0.32	0.62	−3.07	−4.43*	−5.69
School (junior or high)	1.91	2.22	0.17	0.65	0.45	2.91
Tertiary education	3.09	4.14	0.09	0.62	0.03	−6.97
HcY	−0.04	0.17	0.08*	−0.25	−0.19	−1.62
Exercise Group	−1.55	−0.32	0.19	1.40	1.11	2.70
Diet group	−6.78	−3.56	0.51	−1.73	−2.79	15.14
Exercise and diet group	2.17	0.58	0.48	3.48	2.38	2.77
Group effect size (η^2^)	0.07	0.05	0.03	0.05	0.10	0.02

^1^Values were controlled for the corresponding baseline measure, age, sex, education and baseline homocysteine concentrations (unstandardized coefficients). * *P* < 0.05, ***P < 0.001. AIx, augmentation index (AIx = AP/PP × 100); AP, augmentation pressure; Diastolic, peripheral diastolic blood pressure; HcY, homocysteine at baseline; PWV, pulse-wave velocity; PP, central pulse pressure; Systolic, peripheral systolic blood pressure.

### Blood biomarker assessments

Table 5 results indicate that the exercise and diet group had significantly higher concentrations of vitamin B-12 than the control group (*P* < 0.05, η^2^ = 0.07), and in the exercise group vitamin B-6 was significantly lower than for the control group (*P* < 0.05, η^2^ = 0.07) at 6 mo.

**TABLE 5:** Group comparison for biomarker assessment with control group as the reference group^1^

				Vitamin			HbA1c, % range			
Outcomes	TC	GF	hsCRP	B-12	D	B-6		HcY	IGF-I	BDNF
Baseline measure	0.92***	7.21	0.23	0.77***	0.84**	0.66***	0.96***	0.39***	0.67***	0.82***
Age	0.001	0.43	0.04	4.69	0.54	−0.19	−0.00	0.06	−0.01	99.90
Male	−0.25	12.18	−1.64	+0.81	20.90	1.72	0.05	−0.29	0.31	−388.92
School (junior or high)	0.57	−10.85	−6.82**	43.99	2.05	14.53	0.17	0.67	0.36	−4619.19
Tertiary education	0.59	−53.04	−6.85**	51.25	7.36	−9.99	0.06	1.40	0.29	−4733.67
HcY	−0.02	6.0	−0.32*	−0.04	−1.45	1.29	0.00	0.10	0.16	−72.04
Exercise group	−0.03	−81.12	2.20	−3.19	−13.03	−28.88*	−0.20	2.15	−0.20	−1386.52
Diet group	−0.55	24.41	−0.80	−9.23	−11.32	−17.17	0.14	1.04	0.17	−1848.35
Exercise and diet group	−0.09	18.04	−1.47	110.11*	−10.76	−12.59	−0.17	−1.05	0.11	−918.03
Group effect size (η^2^)	0.05	0.09	0.07	0.10	0.02	0.02	0.18	0.10	0.00	0.02

^1^Values were controlled for the corresponding baseline measure, age, sex, education and baseline homocysteine concentrations (unstandardized coefficients). *P < 0.05, **P < 0.01, ***P < 0.001. BDNF, brain-derived neurotrophic factor; GF, fasting glucose; HbA1c, glycated hemoglobin; HcY, homocysteine at baseline; hsCRP, high-sensitivity C-reactive protein; IGF-I, insulin-like growth factor I; TC, total cholesterol.

In addition to biomarker differences between the groups, there were a number of significant associations with control variables. Individuals with school and tertiary education as opposed to postgraduate education had significantly lower concentrations of CRP (*P* < 0.001; η^2^ = 0.17 and 0.15, respectively). In addition, higher homocysteine concentrations were associated with lower concentrations of CRP (*P* < 0.05, η^2^ = 0.09).

### Compliance

From the initiation of the trial, every person allocated to the MedDiet group was requested to complete a daily assessment of how well they believed they had complied with the MedDiet plan by placing a number in a chart each day. Each number represented a compliance percentage, with 1 = <50%, 2 = 50–75%, 3 = 75–90%, and 4 = ≥90%. For participants who completed 6 mo with the MedDiet, 13.9% had an average of 1, 44.4% an average of 2, 33.4% an average of 3, and 8.3% an average of 4. There was a median value of 2 (SD = 0.83), suggesting a 50–75% compliance rate, on average.

Every participant within the walking groups was issued a pedometer and requested to register steps, time, and distance walked per day. A number of issues, such as replacement of pedometers and inaccurate recordings and times, made a compliance estimate difficult to assess. The improvement in walking distance, as assessed by the 6MWT, was the indicator for improvement.

**Table 6** shows the mean changes in 6MWT and MedDietS for each of the groups over 6 mo. However, no significant group differences were found at 6 mo for the 6MWT [*F*(3,72) = 0.536, *P* = 0.659] or the MedDietS [*F*(3,73) = 0.329, *P* = 0.804), when an ANCOVA was conducted controlling for baseline values.

**TABLE 6:** Mean changes in 6MWT and MedDietS for each of the groups over 6 mo^1^

Groups	Change in 6MWT, m	Change in MedDietS	*n*
Control	−5.94 (44.32)	−0.50 (1.41)	24
Exercise	21.11 (41.10)	0.50 (1.25)	20
Diet	7.84 (54.31)	0.24 (1.50)	17
Exercise and Diet	22.31 (84.33)	0.06 (1.34)	17

^1^Values in parentheses are standard deviations. m, meters walked; MedDietS, Mediterranean diet score; 6MWT, 6-Minute Walk Test.

## Discussion

The primary aim of the current study was to examine the 6-mo cognitive performance effects of change to a MedDiet or increased exercise through walking, or a combination of both, in an elderly population living independently in aged-care facilities (23).

As shown in Table 2, contrary to expectations, the primary CompM outcome did not show any significant differences between any of the intervention groups and the control group. However, those in the combined exercise and diet group demonstrated a significant improvement in SWM performance relative to controls. As neither the MedDiet nor exercise interventions alone were associated with SWM performance improvement, it may be that the individual lifestyle interventions were not sufficiently effective in isolation to alter the modifiable risk factors within this time frame. However, the results found with the combined diet and exercise group may be due to additive and/or synergistic aspects of the individual interventions impacting on the mechanistic pathways to improve SWM.

SWM may be associated with a number of lifestyle factors. The neural underpinnings of SWM involve a network of regions including the medial prefrontal cortex and the hippocampus (40). These regions, particularly the hippocampus, are vulnerable to age-associated cognitive decline and, in particular, neurological disorders such as AD (41). Modifying risk factors such as cardiovascular function and oxidative stress, which are targets for lifestyle interventions, has been shown to impact both cognitive function and the structural integrity of the brain (42). Through a regimen of improved lifestyle it is possible that influencing these modifiable risk factors may, in turn, have altered brain structure and/or function, ultimately influencing SWM. More speculatively, these lifestyle factors may have influenced brain plasticity. A comprehensive review has shown that diet modulation has functional implications, resulting in improved adult hippocampal neurogenesis (42).

For all cognitive assessments other than SWM, once the effects of the control variables were taken into account, there were no significant differences between the control group and any of the intervention groups. This is consistent with many previous studies using the SUCCAB cognitive test battery in older individuals, identifying SWM as the most sensitive cognitive domain relevant to age-associated decline (5) and interventions (43). The lack of change in other cognitive domains may also indicate that these domains may require a longer intervention period or that greater compliance to lifestyle change is necessary to identify significant changes, together with greater numbers of participants.

In our secondary outcomes we assessed the effects on mood, quality of life, and overall perceived wellness in these groups (Table 3). We identified that both the exercise group and exercise and diet groups showed a significant reduction in total DASS score. This indicates an improvement in mood, specifically on a combined depression, anxiety, and stress score. This finding is consistent with numerous studies within the extant literature that have found that a change to healthier dietary and exercise regimens is associated with improvements in mental health (44–46).

Conversely, one particularly unexpected result was in the measure of the perception of wellness. The exercise and diet intervention group demonstrated a lower PWS compared with the control group. It is not clear why self-reported wellness was reduced in the exercise and diet intervention group, particularly given that SWM and DASS scores improved in this group. It may be that participants become more aware of their wellness through participation in the trial, with a lower score at 6 mo reflecting a realization that health and self-management of health could be better.

Blood pressure and other vascular health measures have previously been associated with cognitive performance in other studies. However, as shown in Table 4, there were no significant differences between any of the intervention groups and the control group, indicating that the improvement in SWM in the combined intervention group may not be due to vascular changes. Alternatively, the relatively short time frame and group sizes may have limited the chance to detect any change, particular in conjunction with the use of medications, which may have inhibited the expression of change or masked any improvement that may have occurred (47).

Further to this, there was also little change in blood biomarkers in this study, with the only significant changes being an increase in vitamin B-12 in the combined exercise and diet group and a decrease in vitamin B-6 in the exercise-only group. This may be due to the diet-specific components of the intervention or a potential synergistic effect, with increased exercise and better diet resulting in improved absorption of vitamin B-12. The decrease in vitamin B-6 in the exercise-only group is anomalous and the interpretation of this is currently unclear.

The study is one of very few that deal with an older, at-risk group, free from cognitive impairment. Cognition is the primary outcome, applying computerized tests sensitive to age-associated cognitive decline. The group of participants were independently living and residing in supported accommodation and retirement villages, individuals potentially capable of modifying their own dietary and exercise routines. Moreover, the participants were generally not excluded because of the medications they were taking in order to normalize their medical ailments. A strength of the trial was therefore the relevance of the group to the general population, in a setting where lifestyle interventions can potentially be applied to limit the transfer to facilities where a higher level of support is needed, such as aged-care facilities and dementia care. More evidence is needed to provide justification for governments to support lifestyle interventions that target modifiable risk factors to limit cognitive decline and potentially prevent or at least delay the onset of dementia.

Another strength of the trial was that participants were tested in their natural environment. Testing was conducted within these facilities, negating the need for participants to travel to university test laboratories. The logistical and mental challenges associated with travel to, and testing within, unfamiliar surroundings potentially confound cognitive and other results, as well as having impacts on longer-term compliance.

Notwithstanding this advantage of testing within independent-living facilities, there were other impacts on the study that significantly affected study compliance, both in completion of testing at 6 mo and in the ability of participants to comply to the diet and/or exercise-modification requirements.

Potentially, there was a lack of power for detecting significant changes in the subgroups: lower numbers than anticipated at 6 mo and lack of compliance may have reduced the potential for detecting any significant changes with respect to each intervention. In relation to our published protocol (23), we initially estimated the sample size (148), which was not met with only 102 participants recruited at baseline. Additionally, although we predicted a 15% attrition rate, the actual rate was higher than anticipated at 20.5%, resulting in 81 participants remaining at the conclusion of the trial. The reasons participants gave for leaving the trial varied from their inability to maintain the intervention, the incapacity and illness of a partner or loved one, family commitments, and a reduction in their health status. Additional recruitment in future studies (>25%) may be required to ensure that there is adequate power for analysis at the conclusion of a similar type of trial.

As this research was a university-based pilot trial, conducted with limited financial support, there were constraints on resources to support participants with education and motivation in order to maintain their interventional compliance. This may have been another factor for attrition, with participants finding it difficult to adhere to the interventions or losing interest. Moreover, a lack of adherence to the intervention as seen in nonsignificant change scores for the 6MWT and MedDietS relative to control, would have limited efficacy on cognitive and secondary outcomes. Future studies should consider the inclusion of specialist support for both the dietary and exercise regimens throughout the intervention period. This would enhance adherence and long-term compliance and help reduce attrition rates. Additionally, given that it is not possible to blind such interventions, it may be of value in future studies to consider cluster randomization by trial sites as a way to reduce the chance of cross-contamination between groups.

The overall results of this study had multiple tests performed at the 5% significance level. The results must therefore be interpreted cautiously. In addition, no transformations have been applied. This is appropriate in the case of cognitive variables as this allows for easier interpretation. Furthermore, we have not considered medication use in our analyses, as medications were held constant during the trial period. We have previously demonstrated the importance of medication use in the same cohort in assessing relations between diet and cognition (47) Controlling for medication use at baseline may have allowed health factors to be controlled for, together with both the positive and the negative effects of medication use (48). However, the relatively small sample size meant that there was limited scope to also control for a large number of medications.

Compliance with exercise was also difficult due to the inability to have an exercise-monitoring system that would ensure compliance. Participants used their pedometer as a way to encourage them to maintain and then increase their walking. However, this approach is too inaccurate to assess specific walking distances and longer-term walking compliance. Future studies should consider using accelerometers for accurate measurement of physical activity pre- and postintervention and pedometers/smart watches specifically for motivational purposes.

In conclusion, the primary outcome, the CompM, did not show a significant change in any of the 3 intervention groups relative to the control group; however, there was a significant improvement in SWM in the combined diet and exercise group relative to the control group. In addition, this combined exercise and diet group also demonstrated a reduction in negative mood symptoms as assessed by the DASS score over the trial, as did the exercise-only group. This suggests that the interventions, or at least the exercise component, may also improve mood. There were no significant cardiovascular effects and few blood biomarker changes.

Given the number of measures assessed and the small sample size, any interpretation of potential mechanisms from this study would be speculative. We therefore suggest that a study with more participants over a longer time frame is required to further investigate our finding that a combined MedDiet and walking intervention may improve SWM and mood in older people.

With the proliferation of aged-care and retirement facilities within Australia, strategies to keep residents both physically and cognitively healthy are of paramount importance. It is critical to have more evidence-based research to substantiate efficacy, thereby supporting such intervention programs.

## Supplementary Material

nzaa077_Supplemental_File

## Notes

No external financial funding for this research was received. Support in kind was given by Cobram Estate for the supply of extra-virgin olive oil. Dorevitch Pathology provided in-kind support for the storage and collection of bloods. Australian Unity, Villa Maria Catholic Homes, Baptcare, and Churches of Christ Retirement Villages provided the access to independent-living age-care sites in Melbourne. Health Care 2 U provided in-kind preparation for the Mediterranean Diet Book and meal plans.

Author disclosures: The authors report no conflicts of interest.

Supplemental Appendix is available from the “Supplementary data” link in the online posting of the article and from the same link in the online table of contents at https://academic.oup.com/cdn/.

Abbreviations used: AD, Alzheimer disease; AP, augmentation pressure; BDNF, brain-derived neurotrophic factor; CM, contextual memory; CompM, composite memory measure; CRT, choice reaction time; DASS, Depression Anxiety Stress Scale; DRM, delayed recognition memory; EVOO, extra-virgin olive oil; FFQ, food-frequency questionnaire; hsCRP, high-sensitivity C-reactive protein; IRM, immediate recognition memory; LIILAC, Lifestyle Intervention in Independent Living Aged Care; MedDiet, Mediterranean diet; MedDietS, Mediterranean diet score; POMS, Profile of Mood States; PWS, perceived-wellness score; PWV, pulse-wave velocity; SUCCAB, Swinburne University Computerized Cognitive Assessment Battery; SWM, spatial working memory; 6MWT, 6-Minute Walk Test.
