# Supplementary material for: Findings of a Pilot Study Investigating the Effects of Mediterranean Diet and Aerobic Exercise on Cognition in Cognitively Healthy Older People Living Independently within Aged-Care Facilities: The Lifestyle Intervention in Independent Living Aged Care (LIILAC) Study
Source: Curr Dev Nutr. 2020 Apr 18;4(5):nzaa077. doi: 10.1093/cdn/nzaa077 (PMC7228438; doi:10.1093/cdn/nzaa077)
Supplement: nzaa077_Supplemental_File [file nzaa077_supplemental_file.docx]

**Supplementary Appendix: Baseline Comparison of Groups**

.

|  | **Mean (Standard Deviations)** | | | | | |
| --- | --- | --- | --- | --- | --- | --- |
| **Variables** | **Exercise** | **Diet** | **Diet & exercise** | **Control** | **P value** | **η^2^** |
| **Cognitive measures (performance)** |  |  |  |  |  |  |
| Simple reaction time | 33.89(5.27) | 34.57(6.72) | 34.49(5.05) | 34.17(5.44) | 0.97 | 0.002 |
|  |  |  |  |  |  |  |
| Choice reaction time | 19.73(3.11) | 21.32(2.94) | 20.25(2.28) | 19.82(3.22) | 0.20 | 0.46 |
|  |  |  |  |  |  |  |
| Immediate recognition | 6.08(1.86) | 6.45(1.97) | 6.54(1.65) | 6.62(1.59) | 0.74 | 0.14 |
|  |  |  |  |  |  |  |
| Delayed recognition | 6.02(1.51) | 6.50(1.41) | 6.36(1.52) | 6.46(1.37) | 0.63 | 0.18 |
|  |  |  |  |  |  |  |
| Congruent Stroop | 10.91(1.54) | 11.83(1.65) | 12.09(2.27) | 11.21(1.60) | 0.75 | 0.68 |
|  |  |  |  |  |  |  |
| Incongruent Stroop | 8.12(2.01) | 9.03(2.02) | 8.67(2.73) | 7.94(2.09) | 0.27 | 0.39 |
|  |  |  |  |  |  |  |
| Spatial working memory | 4.88 (1.37) | 4.80(1.13) | 5.71(2.02) | 4.73(1.49) | 0.09 | 0.62 |
|  |  |  |  |  |  |  |
| Contextual memory | 6.17(2.37) | 6.59(1.90) | 6.25(1.91) | 6.42(1.99) | 0.89 | 0.006 |
|  |  |  |  |  |  |  |
| **Mood & wellness** |  |  |  |  |  |  |
| Depression | 4.0(5.13) | 1.49(2.19) | 2.2(3.21) | 2.3(3.3) | 0.107 | 0.07 |
|  |  |  |  |  |  |  |
| Anxiety | 3.7(5.2) | 1.1(1.6) | 2.8(3.7) | 2.0(2.86) | 0.102 | 0.07 |
|  |  |  |  |  |  |  |
| Stress | 2.5(3.6) | 1.3(1.9) | 1.5(1.9) | 1.8(2.6) | 0.44 | 0.03 |
|  |  |  |  |  |  |  |
| Total DASS | 7.6(8.5) | 5.8(3.1) | 6.7(4.9) | 3.8(2.5) | 0.067 | 0.08 |
|  |  |  |  |  |  |  |
| PWS | 12.1(2.0) | 12.2(2.0) | 12.0(2.2) | 12.7(1.8) | 0.54 | 0.024 |
|  |  |  |  |  |  |  |
| Total Mood | 12.4(34.9) | 2.7(13.1) | 6.1(23.6) | 0.22(13.7) | 0.29 | 0.41 |
|  |  |  |  |  |  |  |
| **Cardiovascular** |  |  |  |  |  |  |
| Systolic | 138(17.4) | 134(16.8) | 133(12.2) | 137(16.9) | 0.70 | 0.015 |
|  |  |  |  |  |  |  |
| Diastolic | 71.6(11.1) | 69.3(11.0) | 68.2(8.2) | 72.0(10.6) | 0.50 | 0.024 |
|  |  |  |  |  |  |  |
| PWV | 12.0(2.4) | 11.4(1.83) | 11.3(1.68) | 11.3(1.74) | 0.52 | 0.024 |
|  |  |  |  |  |  |  |
| AP | 15.7(7.3) | 14.76.28) | 15.3(6.26) | 16.7(7.54) | 0.779 | 0.011 |
|  |  |  |  |  |  |  |
| AIx | 30.4(10.5) | 28.1(7.34) | 29.8(10.1) | 31.6(11.4) | 0.64 | 0.17 |
|  |  |  |  |  |  |  |
| PP | 53 (11.7) | 49.5 (10.6) | 50.1 (11.3) | 50 (10.1) | 0.75 | 0.016 |
| **Biomarkers** |  |  |  |  |  |  |
| Total cholesterol | 4.84(1.28) | 5.05(1.12) | 4.72(0.73) | 4.60(0.79) | 0.44 | 0.29 |
| mmol/L |  |  |  |  |  |  |
| Glucose fasting | 5.85(1.78) | 5.36(0.52) | 5.87(1.12) | 5.66(0.86) | 0.53 | 0.026 |
| nmol/L |  |  |  |  |  |  |
| CRP | 2.58(3.46) | 3.09(4.65) | 5.25(8.31) | 2.19(4.29) | 0.24 | 0.046 |
| mg/L |  |  |  |  |  |  |
| Vitamin B12 (x100) | 2.99(1.28) | 3.53(1.83) | 3.39(1.71) | 3.14(1.28) | 0.61 | 0.020 |
| pmol/L |  |  |  |  |  |  |
| Vitamin D | 59.2(16.2) | 62.5(22.4) | 61.9(21.7) | 66.0(22.4) | 0.71 | 0.015 |
| nmol/L |  |  |  |  |  |  |
| Vitamin B6 | 32.3(25.2) | 48.9(54.5) | 63.4(50.9) | 62.5(72.8) | 0.17 | 0.055 |
| pmol/L |  |  |  |  |  |  |
| Homocysteine | 17.3(6.36) | 12.3(4.19) | 15.2(5.59) | 15.3(3.81) | 0.009 | 0.12 |
| umol/L |  |  |  |  |  |  |
| HBA1c | 5.76(0.64) | 5.7(0.42) | 5.8(0.49) | 5.6(0.42) | 0.59 | 0.021 |
| mmol/mol |  |  |  |  |  |  |
| IGF1 | 14.1(6.0) | 15.5(4.1) | 18.3(5.6) | 16.2(5.3) | 0.83 | 0.071 |
| nmol/L |  |  |  |  |  |  |
| BDNF (x1000) | 27.5(7.5) | 27.7(11.21) | 30.4(13.0) | 29.5(11.2) | 0.77 | 0.013 |
| pg/ml |  |  |  |  |  |  |
